# Bilateral synchronous spermatocytic seminoma: a rare case report from rural India and literature review

**Published:** 2012-10-17

**Authors:** Sanjay N Koppad, Satish R Sonawane, Vaibhav B Kapoor, Amar M Deshmukh, Ketan A Borole

**Affiliations:** 1 Department of General Surgery, Rural Medical College, Pravara Medical Trust, Loni, Maharashtra, Pin Code – 413736, India

**Keywords:** Bilateral spermatocytic, seminoma, orchidectomy, testicular cancer, germ cell tumour

## Abstract

Spermatocytic Seminoma is an unusual germ cell tumour known to arise from testis only. It is associated with good prognosis. Testicular tumours as such are uncommon in Asia as compared to western countries. In the literature only five cases of bilateral synchronous Spermatocytic Seminoma have been reported. Fifty years male patient presented to us with bilateral scrotal swelling and evaluation revealed neoplastic aetiology of bilateral testicular enlargement. Left side radical orchidectomy was performed initially which histopathologically revealed spermatocytic seminoma. Subsequently right side radical orchidectomy was performed after intra-op frozen section confirmation of neoplastic nature. Histopathology revealed same pathology as on left side. Immunohistochemistry of specimen from both testes was again conclusive of spermatocytic seminoma. We hereby report this rare case of Bilateral Synchronous Spermatocytic Seminoma. This is the first case report from entire Asian continent except for Japan.

## Introduction

Spermatocytic seminoma is an uncommon germ cell neoplasm. It accounts for less than 1% of all testicular tumours and usually arises in older men with a mean age of 53.6 years [1]. Unlike other germ cell tumours it affects only testis. It is characterized by long duration of symptoms with absence of metastasis. Only five cases of bilateral synchronous spermatocytic seminoma have been reported in literature (pubmed database) two each from Japan and France, and one from United States. Hence it is a very rare presentation of this unusual tumor.

## Patient and observation

**Figure 1:**
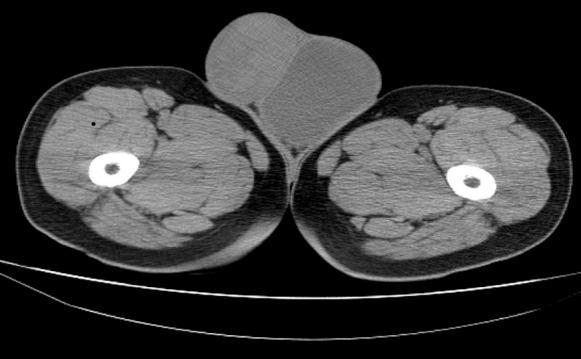
CT image showing bilateral neoplastic testicular enlargement

**Figure 2:**
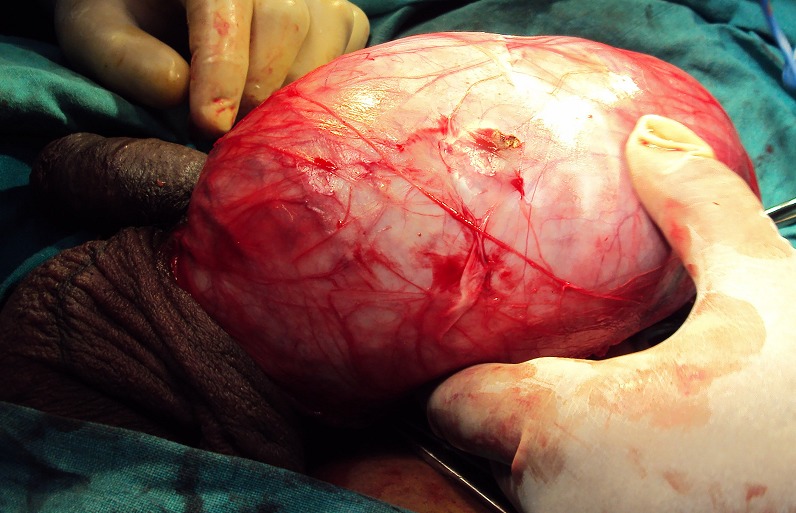
Left inguinal/radical orchidectomy

**Figure 3:**
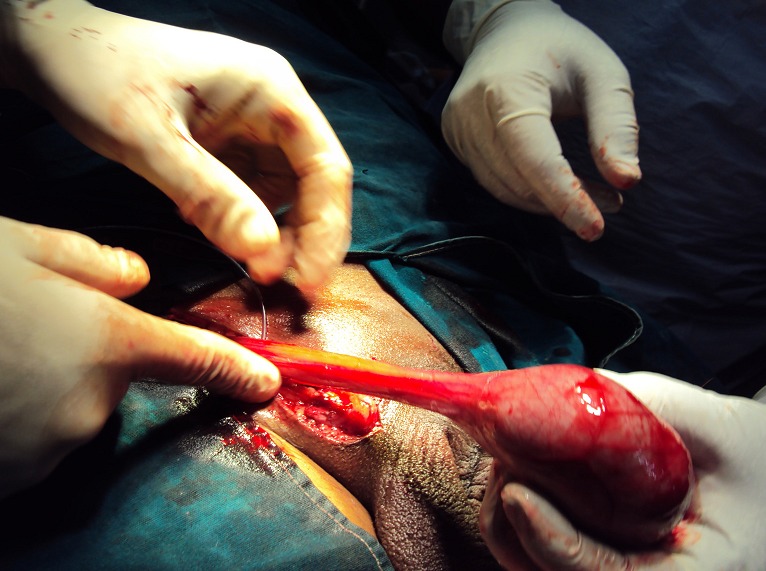
Right inguinal exploration and application of soft clamp before taking frozen section from right testis

**Figure 4:**
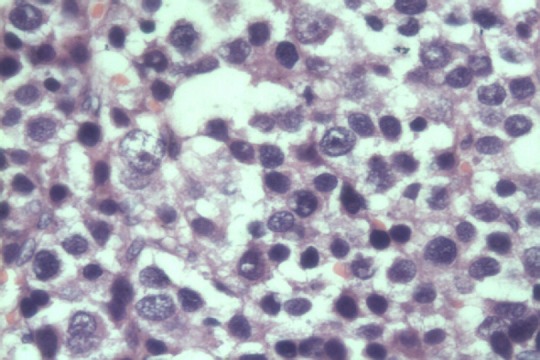
Histopathology picture hematoxylin and eosin stained and 400x magnification with predominant intermediate cell type spermatocytic seminoma

Fifty years male presented with bilateral scrotal swelling. Left side was larger and noticed since five years, and Right side since one and half year. On examination, left testis measured 13 x 8 x 6 cm^3^, non tender, non fluctuant, transillumination negative and uniformly firm in consistency. Testicular sensation was typically absent. Right testis measured 5 x 4 x 3 cm^3^, with similar palpatory findings as left testis. Bilateral groin examination, per abdomen and systemic examination was normal. Ultrasound (USG) scrotum showed bilateral testicular neoplasm with increase vascularity and altered echogenicity and bilateral minimal hydrocele. Ultrasound of abdomen was normal. Tumor markers viz. alpha fetoprotein (AFP), beta HCG, lactate dehydrogenase (LDH) were within normal limits. CT scan abdomen and pelvis showed uniformly enlarged both testis (Figure 1) and no evidence of any retroperitoneal lymphadenopathy. Patient underwent left sided radical/inguinal orchidectomy initially (Figure 2). Histopathology revealed spermatocytic seminoma. Subsequently right sided inguinal exploration (Figure 3) was done and intra-op frozen section biopsy taken from right testis had features of malignant lesion. In view of this, right radical orchidectomy was done. Histopathology of right testis had features similar to left testis thus confirming bilateral spermatocytic seminoma (Figure 4). The histopathological findings were further confirmed on immunohistochemistry, which typically showed absence of placental alkaline phosphates antigen. Patient's on regular follow up, and at the end of ten months there is no evidence of recurrence or metastases.

## Discussion

Testicular tumours are uncommon in Asia compared to Western countries. In Asia, the incidence of testicular tumours is as low as 0.4 per 100,000 population [2]. Spermatocytic seminoma accounts for less than 1% of all testicular tumours. Although classified as a variant of seminoma and first described by Masson [3], in reality they represent an entirely separate and distinct clinicopathological entity, The usual age at diagnosis in spermatocytic seminoma is 50 years in comparison to 30 - 40 years age for classical seminoma. Clinical presentation is usually in the form of a painless testicular swelling or long standing hydrocele [1]. Pure spermatocytic seminoma is an indolent neoplasm and is associated with an excellent prognosis, however with the presence of the sarcomatous component usually rhabdomyosarcoma or undifferentiated /high grade sarcoma, this innocuous neoplasm transforms into an aggressive neoplasm, with most of the patients dying early due to wide spread metastases [4]. Histologically the most distinctive feature of spermatocytic seminoma is cellular variation. Cells with uniformly round nuclei but with variation in diameter (6 – 100 µm (micron)) are distributed in three distinctive populations, small lymphocyte like cells ( 6 – 8 µm) with smudged chromatin and slightly more cytoplasm than a lymphocyte, intermediate sized cells ( 15 – 20 µm), the most common cell type, and large cells ( 50 – 100 µm), that may be mono - or multinucleated. The chromatin is dense in small cells and filamentous in the intermediate and large ones, with a "spireme " appearance, reminiscent of primary spermatocytes, hence the name spermatocytic seminoma. The mitotic rate is often high and apoptosis is prominent. The cytoplasm is eosinophilic to amphophilic and lacks glycogen ( periodic acid Schiff negative). The controversial and rare " anaplastic " variants of spermatocytic seminoma consist of a relatively monomorphic population of intermediate sized cells with only foci of typical spermatocytic seminoma elsewhere [5]. Looijenga et al, had presented data demonstrating that spermatocytic seminomas have a distinct pathogenesis from classical seminomas and non seminomatous germ cell tumours, and are most likely originating from primary spermatocytes. Genome wide analyses of genomic changes and expression profiling confirm the origin of spermatocytic seminoma from primary spermatocytes that have at least initiated prophase meiosis. Therefore the published results indicate that structural chromosomal changes are rare, and that gain of chromosome nine is the only recurrent imbalance [6]. Although spermatocytic seminoma is not associated with intra-tubular germ cell neoplasia (IGCNU), intra-tubular spread is a common finding [7].

It is well known that spermatocytic seminoma is exclusively a testicular tumor, which has never been observed in ovarian or ectopic testis. Spermatocytic seminoma is not associated with any known risk factors for germ cell tumors like cryptorchidism, infertility or gonadal dysgenesis [8]. The metastatic potential of spermatocytic seminoma is extremely low. Reviews by Thackray and Crane (1976) [9] and Weitzner (1979) [10] documented no cases of metastatic disease and prognosis is accordingly favourable. When histologic and staging evaluations have confirmed the diagnosis and the fact that disease is limited to the testis, Treatment beyond inguinal orchidectomy appears unwarranted [9].

## Conclusion

Testicular malignancy as such are unusual. Spermatocytic seminoma is a rare malignancy, and its synchronous bilateral presentation is the rarest. It should be considered, especially while evaluating germ cell tumor in older men. It is distinct in its histologic appearance with three different cell types, lack of cytoplasmic glycogen and sparse or absent lymphocytic infiltrate. Since it rarely metastasizes, correct histologic diagnosis and treatment i.e. orchidectomy can have great impact on prognosis.
